# Use and Misuse of Alcohol Among Older Women

**Published:** 2002

**Authors:** Frederic C. Blow, Kristen Lawton Barry

**Affiliations:** Frederic C. Blow, Ph.D., is an associate professor and senior associate research scientist in the Department of Psychiatry, University of Michigan, Ann Arbor, Michigan, and director, Department of Veterans Affairs National Serious Mental Illness Treatment Research and Evaluation Center, Ann Arbor, Michigan. Kristen Lawton Barry, Ph.D., is a senior associate research scientist in the Department of Psychiatry, University of Michigan, Ann Arbor, Michigan, and associate director, Department of Veterans Affairs National Serious Mental Illness Treatment Research and Evaluation Center, Ann Arbor, Michigan

**Keywords:** alcohol use disorder in the elderly, female, risk factors, protective factors, prevalence, identification and screening for AODD (alcohol and other drug disorders), brief intervention, prevention goals, treatment method, treatment outcome, recommendations or guidelines

## Abstract

Older women may be especially at risk for alcohol problems because they are more likely than men to outlive their spouses and face other losses that may lead to loneliness and depression. Physiologically, women are also at greater risk for alcohol-related health problems as they age. Because of these risks, alcohol use recommendations for older women generally are lower than those set for both older men and younger women. Screening and brief intervention may be especially useful in minimizing alcohol problems in older women. Although brief intervention research with this population is limited, the findings are promising.

The growth in the number of people age 60 and older will bring a soaring increase in the amount and cost of primary and specialty care for this group. In 1990, those over the age of 65 comprised 13 percent of the U.S. population; by the year 2030, older adults are expected to account for 22 percent of the population (U.S. Bureau of the Census 1996). Community surveys have estimated the prevalence of problem drinking among older adults to range from 1 percent to 15 percent (Adams et al. 1996; Fleming et al. 1999; Moore et al. 1999). Among older women, the prevalence of alcohol misuse ranged from less than 1 percent to 8 percent in these studies. As the population age 60 and older increases, so too could the rate of alcohol problems in this age group. However, early detection efforts by health care providers can help limit the prevalence of alcohol problems and improve overall health in older adults.

Many of the acute and chronic medical and psychiatric conditions that lead to high rates of health care use by older people are influenced by the consumption of alcohol. These conditions include harmful medication interactions, injury, depression, memory problems, liver disease, cardiovascular disease, cognitive changes, and sleep problems (Gambert and Katsoyannis 1995). For example, Thomas and Rockwood (2001) found that the occurrence of all types of dementia (with the exception of Alzheimer’s disease) was higher in a sample of 2,873 people age 65 and older with definite or questionable alcohol abuse^1^ compared with those who did not abuse alcohol. At 18 months after baseline, mortality from all causes in this sample was higher among those with definite abuse (14.8 percent) or questionable abuse (20 percent) than among those with no alcohol abuse history (11.5 percent). The risk for negative alcohol-related health effects is greater for older women than for older men at the same amounts of alcohol use.

Researchers have recently recommended that screening and interventions focused on lifestyle factors, including the use of alcohol, may be the most appropriate way to maximize health outcomes and minimize health care costs among older adults (Blow 1998; Barry et al. 2001). For example, primary health care providers can screen patients for alcohol problems and offer brief intervention—5- to 15-minute sessions of information and advice about the risks of drinking and how to reduce drinking—to help prevent at-risk drinkers from developing alcohol problems. In randomized clinical trials, women have been found to benefit most from brief interventions (Fleming et al. 1997, 1999).

This article examines alcohol use among older women, related risk factors and beneficial effects, screening methods to detect alcohol problems in this population, and treatment and prevention approaches.

## Older Women Have Increased Risks for Alcohol Problems

Older women tend to have longer life expectancies and to live alone longer than men, and they are less likely than men in the same age group to be financially independent. These physical, social, and psychological factors are sometimes associated with at-risk drinking in older adulthood, so they are especially relevant for older women.

Older women have major physical risk factors that make them particularly susceptible to the negative effects of increased alcohol consumption (Blow 1998). Women of all ages have less lean muscle mass than men, making them more susceptible to the effects of alcohol. In addition, there is an age-related decrease in lean body mass versus total volume of fat, and the resultant decrease in total body mass increases the total distribution of alcohol and other mood-altering chemicals in the body. Both men and women experience losses in lean muscle mass as they age, but women have less lean muscle mass than men throughout adulthood and, therefore, are less able to metabolize alcohol throughout their lives, including into older adulthood (see Blow 1998 for further information). Liver enzymes that metabolize alcohol and certain other drugs become less efficient with age, and central nervous system sensitivity increases with age for both genders. In sum, compared with younger adults, and with older men, older women have an increased sensitivity to alcohol.

Older women also have a heightened response to over-the-counter and prescription medications (Smith 1995; Vestal et al. 1977; Blow 1998). The use and misuse of alcohol and prescription medications are therefore especially risky for women as they age because of their specific vulnerabilities regarding sensitivity to alcohol and medications. For most patients, any alcohol consumption coupled with the use of specific over-the-counter or prescription medications can be a problem. For example, combining alcohol with psychoactive medications such as benzodiazepines, barbiturates, and antidepressants can be especially problematic for this population. Older women are more likely than older men to receive prescriptions for benzodiazepines in particular, and are therefore more likely to be faced with problems related to the interaction of these medications with alcohol (see Blow 1998 for further discussion). There is a paucity of data available on rates of the co-occurrence of alcohol and medication use in older people. This area needs more study.

Because older women generally drink less than older men or abstain from alcohol, health care providers may be less likely to recognize at-risk drinking and alcohol problems in this population. Moreover, few elderly women who abuse alcohol seek help in specialized addiction treatment settings. These problems stand in the way of effective interventions that can improve the quality of life of older women drinking at risky levels.

The following sections will first examine the prevalence of problem drinking in older women and then review the risks and benefits associated with alcohol use among older women. The article concludes with a discussion of screening and interventions for this population.

## Prevalence of the Problem

As stated above, community surveys have estimated that at-risk drinking ranges from 1 percent to 15 percent among older adults and that from 1 percent to 8 percent of older women misuse alcohol (Adams et al. 1996; Fleming et al. 1999; Moore et al. 1999). The wide variation of these ranges results from varying definitions of problem drinking and alcohol misuse and from the methodology used in selecting the survey respondents.

The rates of illegal drug abuse among the older population are very low. Because of the dearth of information in this area, actual rates are difficult to measure (Blow 1998). Future research will more completely address the use of alcohol and illegal drugs in older adulthood (Blow 1998).

Prescription drug misuse is more common and has multiple determinants, causes, and consequences. For example, older adults may be experiencing problems related to overuse of prescription drugs because they are prescribed more medication than they can tolerate at that age, or because they are seeking prescriptions for a particular medication (e.g. benzodiazepine) from multiple providers.

## Risk and Benefits Associated with Alcohol Use by Older Women

Research provides evidence that one drink^2^ per day (i.e., moderate drinking) is associated with certain health benefits among older adults generally and among older women, whereas higher levels of drinking are associated with health risks. Only a few studies have either focused on women or have included sufficient numbers of older women to be conclusive about the effects for this population (Abramson et al. 2001). Therefore, this section includes studies on older adults in general and on women in particular.

### Risks

A recent study of moderate and heavy drinking among older adults found that study participants reported poorer psychosocial functioning with increasing daily alcohol consumption (Graham and Schmidt 1999). The frequency of drinking (drinking days per week), however, was not related to psychosocial well-being, suggesting that the amount of alcohol consumption was a more significant factor. Ensrud and colleagues (1994) found that, among older women, those with a history of regular alcohol use were 2.2 times more likely to have impaired activities of daily living compared with those with no history of regular alcohol use. Alcohol use was more strongly correlated with impairment than were smoking, age, use of antianxiety medication, or stroke.

Although several studies have examined the role of alcohol use in cardiac problems, stroke, and cancers, most of these studies have not included older women. A study using National Cholesterol Education Program data found that, among the women in the study, failure to use lipid-lowering medications was associated with alcohol consumption and smoking, among other factors (Schrott et al. 1997). In a study of postmenopausal women in the Iowa Women’s Health Study, Sellers and colleagues (2002) estimated the interaction of folate intake from diet and alcohol consumption at baseline for 34,393 study participants to determine the risk for specific types of breast cancer. The study compared women with low folate levels and higher alcohol consumption (i.e., more than 4 grams per day)^3^ with nondrinkers who had a high folate intake. The authors found that the combination of alcohol use and low folate levels produced an increase in the risk of one type of tumor. A recent meta-analysis examined 53 epidemiological studies of the relationships between alcohol use, smoking, and breast cancer (Collaborative Group on Hormonal Factors in Breast Cancer 2002), including 58,515 women with breast cancer and 95,067 women without the disease. This study found that, compared with women who reported drinking no alcohol, the relative risk was 1.32 for those who drank 35 to 44 grams of alcohol per day (3 to 3.6 standard drinks), and 1.46 for those who drank more than 45 grams per day (3.75 standard drinks). A relative risk of 1.32 corresponds to a 32-percent higher risk. The relative risk of breast cancer increased by 7.1 percent for every 10 grams of alcohol consumed per day.

Epidemiological studies have clearly demonstrated that comorbidity between alcohol use and psychiatric symptoms is common in younger age groups. Less is known about comorbidity between alcohol use and psychiatric illness in later life. A few studies have indicated that a dual diagnosis with alcoholism is an important negative predictor of outcomes among the elderly (Blow 1998; Saunders et al. 1991; Finlayson et al. 1988). Because women are twice as likely as men to experience depression, and older women often experience several life losses that can exacerbate depression and the use of alcohol, it is important for health care providers to be aware of the potential for comorbid depression and alcoholism in this population and to keep potential comorbid factors in mind when conducting health screenings with older women, particularly when they are experiencing some of the difficult personal losses associated with aging.

### Benefits

There is growing evidence that, among otherwise healthy adults, especially middle-aged adults, moderate alcohol use may reduce risks of cardiovascular disease (Scherr et al. 1992; Thun et al. 1997), some dementing illnesses, and some cancers (Broe et al. 1998; Orgogozo et al. 1997; Klatsky et al. 1997). Simons and colleagues (2000) found that moderate alcohol intake (from 1 to 14 drinks per week) in older men and women was associated with decreased mortality. Nelson and colleagues (1994) have demonstrated that older people living in the community (not in institutions) who consume moderate amounts of alcohol have fewer falls, greater mobility, and improved physical functioning when compared with nondrinkers. One of the factors affecting the disparities between the results of various studies on this topic may be the setting for the study (e.g., community, subsidized housing, assisted living situation, or institution).

In a meta-analysis of studies of alcohol’s effect on coronary heart disease, Mukamal and Rimm (2001) found that two drinks per day increased high-density lipoprotein (HDL) cholesterol levels, translating to a 16.8-percent decreased risk of coronary heart disease. Additionally, a study of women with coronary heart disease found that older age, alcohol consumption, and prior estrogen use were all independently associated with higher HDL cholesterol (Bittner et al. 2000).

The debate regarding the benefits and liabilities of alcohol use for older women continues. As new studies include larger numbers of older women, definitive recommendations regarding the relationships between alcohol use and cancers, stroke, cardiac diseases, and risk of psychiatric comorbidities will become more feasible.

Based on the risk factors associated with alcohol use by older women, drinking guidelines for this population are lower than those set for other adults, as reviewed in the next section.

## Drinking Guidelines and Rationale

Because of the age-related changes in how alcohol is metabolized and the potential interactions between medications and alcohol, alcohol use recommendations for older adults are generally lower than those set for adults younger than age 65. Recommendations for women are slightly lower than those for men as they age.

The National Institute on Alcohol Abuse and Alcoholism (NIAAA) and the Center for Substance Abuse Treatment (CSAT) (Blow 1998) recommend that people age 65 and older consume no more than one standard drink per day or seven standard drinks per week (Dufour and Fuller 1995).

These recommendations are consistent with the current evidence weighing the risks and beneficial health effects of drinking (Klatsky et al. 1997; Mukamal and Rimm 2001). To put these recommendations into perspective, the guidelines for adults younger than age 65 are as follows: for women, no more than one standard drink per day; for men, no more than two standard drinks per day (U.S. Department of Health and Human Services and U.S. Department of Agriculture 1995).

### Definitions

Before discussing screening and intervention, it is important to define the various levels of drinking. These definitions help anchor clinical decisions regarding when and if interventions are needed. Drinking that exceeds the guidelines will not always lead to alcohol-related problems, particularly for people who are drinking a few drinks above recommended limits but not at levels that can put them at risk for alcohol dependence. It is, however, useful to consider a model indicating that the more alcohol a person consumes, the more likely that person is to have alcohol-related problems (Institute of Medicine 1990). Categories of drinking risk presented here—low-risk drinking, at-risk drinking, problem drinking, and alcohol dependence—are based on that conceptualization and form a framework for understanding the spectrum of use seen in older women (Blow 1998; Barry et al. 2001).

#### Abstinence

Approximately 60 to 70 percent of older adults (70 to 80 percent of older women) abstain from drinking. Reasons for abstinence may include religious beliefs, illnesses, or previous problems with alcohol use. Alcohol-use interviews ascertain the reasons for nonuse.

***Low-risk drinking*** is low-level alcohol use that is not problematic. Older women in this category drink within recommended drinking guidelines (less than one drink per day or seven drinks per week), are able to employ reasonable limits on alcohol consumption, and do not drink when driving a motor vehicle or when using medications that may interact with alcohol.

Low-risk use of medications or other drugs would include using medications following the physician’s prescription. However, screening should include a check on the number and types of medications a person is using and her concomitant use of alcohol, because interactions between medications and alcohol are not uncommon in older women.

***At-risk drinking*** increases the chance that a person will develop drinking-related problems. Women age 65 and older who drink more than one drink per day are in the at-risk use category. Brief advice or brief interventions can be useful for women in this group.

***Problem drinking*** among older women is defined as the consumption of alcohol at a level that has already resulted in adverse medical, psychological, or social consequences. Potential consequences may include injuries, medication interaction problems, and family problems. The presence of consequences, whether or not the person’s drinking exceeds the recommended guideline, also suggests a need for intervention.

***Alcohol abuse and dependence*** are disorders characterized by specific criteria. Alcohol abuse is characterized by continued drinking despite negative consequences and the inability to fulfill responsibilities. Alcohol dependence, also known as alcoholism, is characterized by loss of control, preoccupation with alcohol or other drugs, and physiological symptoms such as tolerance and withdrawal (American Psychiatric Association [APA] 1994). Women age 65 and older who have alcohol abuse or dependence disorders can benefit greatly from treatment, especially elder-specific programs (Blow et al. 2000; Schonfeld et al. 2000).

## Screening and Detection of Alcohol Problems in Older Women

CSAT (Blow 1998) has recommended that everyone age 60 and older should be screened for alcohol and prescription drug use and abuse as part of regular health care services. People should continue to be screened yearly unless certain physical or mental health symptoms emerge during the year, or unless they are undergoing major life changes or transitions, at which time additional screenings should be conducted. The textbox lists some of the signs and symptoms of alcohol problems seen in older women. Many of these signs can be related to other problems that occur in later life, but it is important to rule alcohol use in or out of any diagnosis.

**Signs and Symptoms of Alcohol Problems in Older Women**

- Anxiety
- Increased tolerance to alcohol or medications
- Depression, mood swings
- Memory loss
- Disorientation
- New difficulties in decisionmaking
- Poor hygiene
- Falls, bruises, burns
- Family problems
- Idiopathic seizures (i.e., seizures with an unknown origin or cause)
- Financial problems
- Sleep problems
- Headaches
- Social isolation
- Incontinence
- Poor nutrition

SOURCE: Adapted from Barry et al. 2001.

The goals of screening are to identify at-risk drinkers, problem drinkers, or people with alcohol abuse or dependence disorders and to determine the need for further assessment. Screening can take place in a variety of settings including primary care, specialty care, and social service and emergency departments. Alcohol screening can be conducted because the incidence of alcohol problems is high enough to justify the cost, alcohol can adversely affect morbidity and mortality, and valid, cost-effective screening methods and effective treatments are available.

Systems (e.g., automatic yearly administration of alcohol screening instruments) to ensure that older women in health care settings are screened for alcohol use and consequences are necessary for prevention and early intervention efforts. These systems must include screening for alcohol use (frequency and quantity), drinking-related consequences, medication use and alcohol/medication interaction problems, and depressed feelings. Screening may be conducted as part of routine mental and physical health services and can be updated annually. Screening should also take place before a patient begins taking any new medications or in response to problems that may be related to alcohol or medication.

Clinicians can obtain more accurate patient histories by asking questions about the recent past and by asking the alcohol use questions in the context of other health variables (e.g., exercise, weight, smoking). Alcohol (and other drug) screening for older patients should be simple and consistent with other screening procedures already in place.

Screening for alcohol use and related problems is not always standardized, and not all standardized instruments are reliable and valid with older women. The Short Michigan Alcoholism Screening Test–Geriatric Version (SMAST–G) (Blow et al. 1998), which consists of quantity and frequency questions embedded with questions about other health habits (see Blow 1998 for a review of screening instruments for older adults), and the newer Alcohol-Related Problems Survey (Moore et al. 1999) are both valid and reliable instruments with older adults. The CAGE^4^ (Ewing 1984), a widely used alcohol screening test, does not have high validity with older adults, in particular with older women (Adams et al. 1996).

## Prevention, Brief Intervention, and Formal Treatment with Older Women

For years, screening and brief intervention have been suggested as cost-effective and practical techniques that can be used with at-risk and problem drinkers in primary care settings. CSAT has defined brief alcohol interventions as time limited (from 5 minutes to five brief sessions) and targeting a specific health behavior (at-risk drinking) (Barry 1999). Over the last two decades, more research has evaluated the effectiveness of early problem detection and secondary prevention (i.e., preventing existing problems from getting worse). Such studies have evaluated brief intervention strategies for treating problem drinkers, especially those with relatively mild-to-moderate alcohol problems who are potentially at risk for developing more severe problems (Fleming et al. 1997).

### Brief Alcohol Intervention Goals

Brief intervention typically includes setting flexible drinking goals that allow the patient, with guidance from the clinician, to choose drinking moderation or abstinence. The goal of brief intervention is to motivate at-risk and problem drinkers to change their behavior—that is, to reduce or stop alcohol consumption. In some cases, when formal treatment is warranted, the goal is to facilitate treatment entry. Terminology can be particularly important when working with older women. The stigma and shame associated with the term “alcoholic” can be a powerful deterrent to seeking help. Avoiding pejorative terms provides a positive framework for clinicians and can help empower older women with risky alcohol or medication use to make changes, thereby reducing the negative feelings often associated with drinking problems.

Brief alcohol interventions can be conducted using guidelines and steps (Barry et al. 2001) adapted from work by Wallace and colleagues (1988), Fleming and colleagues (1997), and Blow and Barry (2000). Brief alcohol intervention protocols are designed for busy clinicians and often use a workbook that the patient can take home at the end of the session. Auxiliary issues included in the brief alcohol intervention for older women vary based on individual patient issues and the time available for the intervention.

### Effectiveness of Brief Alcohol Interventions with Older At-Risk Drinkers

The spectrum of alcohol intervention for older adults ranges from prevention/education for abstinent or low-risk drinkers and minimal advice or brief structured interventions for at-risk or problem drinkers to formal alcoholism treatment for drinkers who meet the criteria for alcohol abuse or dependence (Blow 1998). Although referral to formal treatment is appropriate for patients with alcohol abuse or dependence, pretreatment strategies are also appropriate for this population. Pre-treatment strategies include the use of brief interventions to help patients discriminate between their alcohol use and the problems resulting from that use (Barry 1999).

Brief interventions for alcohol problems (for all populations) have employed various approaches to change drinking behaviors. Strategies have ranged from relatively unstructured counseling and feedback to more formal structured therapy (see Barry 1999 for a review) and have relied heavily on concepts and techniques from the behavioral self-control training literature (Miller and Rollnick 1991).

Several brief alcohol intervention studies conducted in primary care settings with younger adults have shown mainly positive results. Both brief interventions and brief therapies (usually delivered by mental health professionals to people in substance abuse or mental health treatment) have been found to be effective in a range of clinical settings including primary care, mental health treatment, hospital, senior housing, and senior centers (Barry 1999). Although fewer studies with older adults are available, two existing studies suggest that brief intervention is useful with the older population as well. Fleming and colleagues (1999) and Blow and Barry (2000) used brief interventions in randomized clinical trials in primary care settings to reduce hazardous drinking among older adults. These studies have shown that older adults can be engaged in brief intervention, that this technique is acceptable in this population, and that there is a substantial reduction in drinking among at-risk drinkers receiving the interventions compared with a control group.

The first study, Project GOAL: Guiding Older Adult Lifestyles (Fleming et al. 1999), was a randomized controlled clinical trial conducted in Wisconsin with 158 older adults ages 65 to 88, 53 (34 percent) of whom were women. All patients age 65 and older in a number of primary care sites were asked to complete a screening questionnaire. Those who screened positive for at-risk drinking (i.e., those who exceeded recommended drinking guidelines) were randomized to an intervention group and a control group. One hundred forty-six subjects participated in the 12-month followup. The intervention consisted of two 10-to 15-minute counseling visits during which the physician delivering the intervention followed a scripted workbook; the patients were given advice and information and asked to sign a contract designed to reinforce drinking goals. At baseline, both groups consumed an average of 15 to 16 drinks per week. After 12 months, patients in the intervention group drank significantly less than those in the control group, decreasing their consumption by about 30 percent. Because the proportion of women in the study was small, major analyses focused on the entire sample of men and women together.

The second elder-specific study, the Health Profile Project, was conducted in primary care settings in southeast Michigan (Blow and Barry 2000). Examining a sample that included patients age 55 and older, researchers sought to determine whether changes in drinking patterns and response to interventions occurred both in older adulthood (older than 65) and in the transitional phase from ages 55 to 65. The older-adult-specific intervention, used with both groups for consistency, included both a brief advice discussion with a psychologist or social worker and motivational interviewing techniques, and feedback. A total of 420 people participated (including those who received the intervention and the control group) in this trial, and 367 participated in 12-month followup interviews. Seventy-three women were enrolled in the study at baseline, and 69 participated in the 12-month followup. The mean age of the female participants was 67.

The study found results similar to the study by Fleming and colleagues (1999) for binge drinking (i.e., drinking four or more drinks per occasion) and drinking days per week, in particular, at 12-month followup. At followup, the intervention group of women averaged 7 drinks per week (within recommended guidelines) and the control group averaged 8.2 drinks per week. Although the intervention group lowered its consumption to within NIAAA guidelines, the groups were not statistically different at followup. Nor did the groups differ significantly in terms of drinks per day at baseline or followup. The fact that the intervention and control group did not differ in drinks per drinking day at 12 months after intervention could indicate natural minimal changes over time in behaviors for both groups. However, there were statistically significant differences between the groups in days per week (frequency) of drinking from baseline to 12 months. On average, subjects in the intervention group decreased their drinking from 4.5 days per week at baseline to 3.1 days per week at 12 months; the control subjects drank an average of 4.3 days per week at baseline and only decreased to 3.6 days per week at 12-month followup. The intervention group showed significantly more days of abstinence per week at 12 months, indicating diminished risk. Days of abstinence are recommended for reducing risk (Barry et al. 2001).

These randomized controlled clinical trials extend the positive results of research on younger at-risk drinkers to the older at-risk drinking population by showing that, regardless of age, brief interventions are effective in assisting older at-risk drinkers to drink less often. The studies provide a good basis for future research focused on older women who use alcohol and on the interaction between alcohol and medications in this age group. Research is needed to determine the most effective components of brief interventions with older women and the most effective venues (e.g., primary care, in-home, senior center, senior housing). Research is also needed to address an under-studied area, the interaction between alcohol and medications in older women, and to determine the best methods for dealing with this more complex problem.

Because the population of older women is increasing rapidly and rates of alcohol misuse are anticipated to increase with the aging of the Baby Boom generation, alcohol researchers need to find methods to include larger numbers of older women in studies. Randomized trials with larger sample sizes will provide a more complete picture of the characteristics of women who respond to brief interventions as well as the most effective education and prevention methods for this population.

## Formal, Specialized Treatment Approaches for Older Women

CSAT has recommended several approaches for the effective formal treatment of older women and men with alcohol problems. These include cognitive behavioral approaches, group-based approaches, individual counseling, medical/psychiatric approaches, marital and family involvement/family therapy, case management/community-linked services and outreach, and formal alcoholism treatment.

As with all other clinical issues, not every approach fits every older woman with alcohol abuse or dependence. Ideally, treatment should be individualized for the specific person, taking into account his or her medical, psychiatric, social, and cultural needs. Most of the therapeutic approaches included here have been more widely studied in younger adults (Blow 1998). Only a few elder-specific studies have evaluated intervention/treatment methods other than brief intervention for at-risk drinkers and formal treatment for people with alcohol abuse or dependence. There has been even less of a focus on older women, in part because fewer older women meet criteria for formal treatment and because fewer women who need treatment are identified by primary providers and referred to treatment. A few examples of elder-specific studies are available, however.

Blow and colleagues (2000) and Schonfeld and colleagues (2000) found that cognitive-behavioral approaches— such as teaching older adults skills necessary to rebuild social support networks and using self-management approaches for overcoming depression, grief, and loneliness—were successful in reducing or stopping alcohol use.

Research has also found that case management services are helpful for older adults receiving alcoholism treatment and may be the best way to provide outreach services. Because traditional residential alcoholism treatment programs generally treat few older adults, small sample sizes have prevented the evaluation of formal treatment. The development of elder-specific alcoholism treatment programs in recent years has identified sufficiently large numbers of older adults with alcohol abuse or dependence disorders to begin to facilitate studies of this population (Atkinson 1995). A remaining limitation with this age group is the lack of longitudinal studies of treatment outcomes.

In one of the few long-term studies of an elder-specific specialized alcoholism treatment program, Blow and colleagues (2000) examined multidimensional 6-month outcomes for 90 patients older than age 55. At baseline, physical health functioning was similar to that reported by seriously medically ill patients (with and without alcohol problems) in other studies, whereas psychological functioning was worse. Nearly one-third of the sample had comorbid psychiatric disorders. Results suggested that the largest percentage of older adults who received elder-specific substance abuse treatment attained positive outcomes and that their conditions improved across a range of physical and psychosocial measures. Further research is needed in this area to determine the following:

- If elder female-specific specialized treatment is necessary, effective, or both
- If older women in elder-specific programs show better outcomes than older women in mixed-age programs
- If intervention and treatment approaches for alcohol and prescription drug misuse are effective with older women.

## Summary

The growing population of older adults reflects the need for new, innovative prevention and intervention techniques and approaches targeted to older at-risk drinkers. These approaches should consider elder-specific characteristics such as alcohol-related symptoms and patterns of use, age of onset, and medical and mental health issues.

The range of prevention and intervention strategies available to older adults—prevention and education for people who are abstinent or low-risk drinkers, minimal advice and brief intervention for at-risk drinkers, and formal treatment for people with alcohol abuse or dependence—provides the necessary tools for health care providers to give high-quality care to older adults across the spectrum of drinking patterns.

Although some progress has been made in understanding the effectiveness of alcohol screening, brief intervention, and treatment among older women, it remains to be determined how these protocols fit into the broad spectrum of health care settings (e.g., primary care, mental health care, specialty physical health care, hospitals) and how to target specific interventions or treatments to appropriate subgroups of older women. The health care field must develop and test time- and cost-effective methods of screening, intervention, and treatment to provide optimal care to a vulnerable, growing, and under-recognized population of older women who are consuming alcohol and other drugs.

## Footnotes

1 Based on interview results and criteria from the *Diagnostic and Statistical Manual of Mental Disorders, Fourth Edition* (DSM–IV) (American Psychiatric Association 1994).

2 A standard drink is 12 ounces of regular beer, 5 ounces of wine, or 1.5 ounces of 80-proof distilled spirits.

3 There are 12 grams of alcohol in a standard drink in the United States.

4 The CAGE screening instrument (Ewing 1984) consists of four questions:

- Have you ever felt you should **C**ut down on your drinking?
- Have people **A**nnoyed you by criticizing your drinking?
- Have you ever felt bad or **G**uilty about your drinking?
- Have you ever had a drink first thing in the morning to steady your nerves or to get rid of a hangover (**E**ye opener)?

## References

[b1-308-315] Abramson JL, Williams SA, Krumholz HM, Vaccarino V (2001). Moderate alcohol consumption and risk of heart failure among older persons. JAMA: Journal of the American Medical Association.

[b2-308-315] Adams WL, Barry KL, Fleming MF (1996). Screening for problem drinking in older primary care patients. JAMA: Journal of the American Medical Association.

[b3-308-315] American Psychiatric Association (APA) (1994). Diagnostic and Statistical Manual of Mental Disorders.

[b4-308-315] Atkinson R, Beresford TP, Gomberg ESL (1995). Treatment programs for aging alcoholics. Alcohol and Aging.

[b5-308-315] Barry KL (1999). Brief Interventions and Brief Therapies for Substance Abuse.

[b6-308-315] Barry KL, Oslin D, Blow FC (2001). Prevention and Management of Alcohol Problems in Older Adults.

[b7-308-315] Bittner V, Simon JA, Fong J (2000). Correlates of high HDL cholesterol among women with coronary heart disease. American Heart Journal.

[b8-308-315] Blow F (1998). Substance Abuse Among Older Adults.

[b9-308-315] Blow FC, Barry KL (2000). Older patients with at-risk and problem drinking patterns: New developments in brief interventions. Journal of Geriatric Psychiatry and Neurology.

[b10-308-315] Blow F, Gillespie BW, Barry KL Brief screening for alcohol problems in elderly populations using the Short Michigan Alcoholism Screening Test–Geriatric Version (SMAST–G).

[b11-308-315] Blow FC, Walton MA, Chermack ST (2000). Older adult treatment outcomes following elder-specific inpatient alcoholism treatment. Journal of Substance Abuse Treatment.

[b12-308-315] Broe GA, Creasey H, Jorm AF (1998). Health habits and risk of cognitive impairment and dementia in old age: A prospective study on the effects of exercise, smoking and alcohol consumption. Australian and New Zealand Journal of Public Health.

[b13-308-315] Collaborative Group on Hormonal Factors in Breast Cancer (2002). Alcohol, tobacco and breast cancer: Collaborative reanalysis of individual data from 53 epidemiological studies, including 58,515 women with breast cancer and 95,067 women without the disease. British Journal of Cancer.

[b14-308-315] Dufour MC, Fuller RK (1995). Alcohol in the elderly. Annual Review of Medicine.

[b15-308-315] Ensrud KE, Nevitt MC, Yunis C (1994). Correlates of impaired function in older women. Journal of the American Geriatrics Society.

[b16-308-315] Ewing JA (1984). Detecting alcoholism: The CAGE questionnaire. JAMA: Journal of the American Medical Association.

[b17-308-315] Finlayson RE, Hurt RD, Davis LJ, Morse RM (1988). Alcoholism in elderly persons: A study of the psychiatric and psychosocial features of 216 inpatients. Mayo Clinic Proceedings.

[b18-308-315] Fleming MF, Barry KL, Manwell LB (1997). Brief physician advice for problem alcohol drinkers: A randomized controlled trial in community-based primary care practices. JAMA: Journal of the American Medical Association.

[b19-308-315] Fleming MF, Manwell LB, Barry KL (1999). Brief physician advice for alcohol problems in older adults: A randomized community-based trial. Journal of Family Practice.

[b20-308-315] Gambert SR, Katsoyannis KK, Beresford TP, Gomberg E (1995). Alcohol-related medical disorders of older heavy drinkers. Alcohol and Aging.

[b21-308-315] Graham K, Schmidt G (1999). Alcohol use and psychosocial well-being among older adults. Journal of Studies on Alcohol.

[b22-308-315] Institute of Medicine (1990). Broadening the Base of Treatment for Alcoholism.

[b23-308-315] Klatsky AL, Armstrong MA, Friedman GD (1997). Red wine, white wine, liquor, beer, and risk for coronary artery disease hospitalization. American Journal of Cardiology.

[b24-308-315] Miller WR, Rollnick S (1991). Motivational Interviewing: Preparing People to Change Addictive Behavior.

[b25-308-315] Moore AA, Morton SC, Beck JC (1999). A new paradigm for alcohol use in older persons. Medical Care.

[b26-308-315] Mukamal KJ, Rimm EB (2001). Alcohol’s effect on the risk of coronary heart disease. Alcohol Research & Health.

[b27-308-315] Nelson HD, Nevitt MC, Scott JC (1994). Smoking, alcohol, and neuromuscular and physical function of older women. JAMA: Journal of the American Medical Association.

[b28-308-315] Orgogozo JM, Dartigues JF, Lafont S (1997). Wine consumption and dementia in the elderly: A prospective community study in the Bordeaux area. Revue Neurologique (Paris).

[b29-308-315] Saunders PA, Copeland JR, Dewey ME (1991). Heavy drinking as a risk factor for depression and dementia in elderly men. British Journal of Psychiatry.

[b30-308-315] Scherr PA, LaCroix AZ, Wallace RB (1992). Light to moderate alcohol consumption and mortality in the elderly. Journal of the American Geriatrics Society.

[b31-308-315] Schonfeld L, Dupree LW, Dickson-Fuhrmann E (2000). Cognitive-behavioral treatment of older veterans with substance abuse problems. Journal of Geriatric Psychiatry and Neurology.

[b32-308-315] Schrott HG, Bittner V, Vittinghoff E (1997). Adherence to National Cholesterol Education Program treatment goals in post-menopausal women with heart disease. The Heart and Estrogen/Progestigen Replacement Study (HERS). JAMA: Journal of the American Medical Association.

[b33-308-315] Sellers TA, Vierkant RA, Cerhan JR (2002). Interaction of dietary folate intake, alcohol, and risk of hormone receptor-defined breast cancer in a prospective study of postmenopausal women. Cancer Epidemiology.

[b34-308-315] Simons LA, McCallum J, Friedlander Y (2000). Moderate alcohol intake is associated with survival in the elderly: The Dubbo study. Medical Journal of Australia.

[b35-308-315] Smith JW (1995). Medical manifestations of alcoholism in the elderly. International Journal of the Addictions.

[b36-308-315] Thomas VS, Rockwood KJ (2001). Alcohol abuse, cognitive impairment, and mortality among older people. Journal of the American Geriatric Society.

[b37-308-315] Thun MJ, Peto R, Lopez AD (1997). Alcohol consumption and mortality among middle-aged and elderly U.S. adults. New England Journal of Medicine.

[b38-308-315] U.S. Bureau of the Census (1996). 65+ in the United States.

[b39-308-315] U.S. Department of Agriculture (USDA) and U.S. Department of Health and Human Services (1995). Nutrition and Your Health: Dietary Guidelines for Americans.

[b40-308-315] Vestal RE, McGuire EA, Tobin JD (1977). Aging and ethanol metabolism. Clinical Pharmacology and Therapeutics.

[b41-308-315] Wallace P, Cutler S, Haines A (1988). Randomized controlled trial of general practitioner intervention in patients with excessive alcohol consumption. British Medical Journal.

